# Supplementary material for: Ultrasound-induced reorientation for multi-angle optical coherence tomography
Source: Nat Commun. 2024 Mar 16;15:2391. doi: 10.1038/s41467-024-46506-2 (PMC10944478; doi:10.1038/s41467-024-46506-2)
Supplement: Supplementary file 3 — Description of Additional Supplementary Files [file 41467_2024_46506_MOESM3_ESM.pdf]

Description of Additional Supplementary Files

Supplementary Movie 1. Animation showing working principle of ULTIMA-OCT (not to scale), rendered in Blender.

Supplementary Movie 2. Recording of reorientation of 3 dpf *Mitfa* $^{b692/b692}$/*ednrb*$^{1b140/b140}$ zebrafish (darkfield imaging) with two transducers by changing voltage and pressure in a step-wise manner.

Supplementary Movie 3. Recording of melanoma spheroid manipulated by two transducers: stably trapped at 4 orientations, undersustained rotation, and change of rotation direction (darkfield imaging).

Supplementary Movie 4. 3D rendering of a 3 dpf *Mitfa* $^{b692/b692}$/*ednrb*$^{1b140/b140}$ zebrafish embryo showing shadow artifacts. Scale bar: 300 µm.

Supplementary Movie 5. Visualization of three dimensional renderings of raw OCM volumes and reconstruction.

Supplementary Movie 6. Flythroughs through raw OCM volumes and reconstruction across three axes.
